# 161. DoxyPEP uptake in a Southeastern HIV and PrEP clinic: missed opportunities and gaps in implementation

**DOI:** 10.1093/ofid/ofaf695.056

**Published:** 2026-01-11

**Authors:** Emily D Niehaus, Justin Frye, Charles M Burns, Nwora Lance Okeke

**Affiliations:** Duke University School of Medicine, Durham, NC; Duke University School of Medicine, Durham, NC; Duke University, Durham, North Carolina; Duke University, Durham, North Carolina

## Abstract

**Background:**

Bacterial sexually transmitted infection (STI) incidence has increased in recent decades, disproportionately affecting young people, Black and Hispanic individuals, and men who have sex with men (MSM). DoxyPEP is a strategy to prevent acquisition of bacterial STIs among high-risk groups, with the Centers for Disease Control (CDC) recommending use in MSM and transgender women (TGW) with a recent STI. We evaluated DoxyPEP prescribing patterns and missed opportunities to prescribe the intervention after a bacterial STI diagnosis in a Southeastern HIV/PrEP outpatient clinic.

**Figure:**
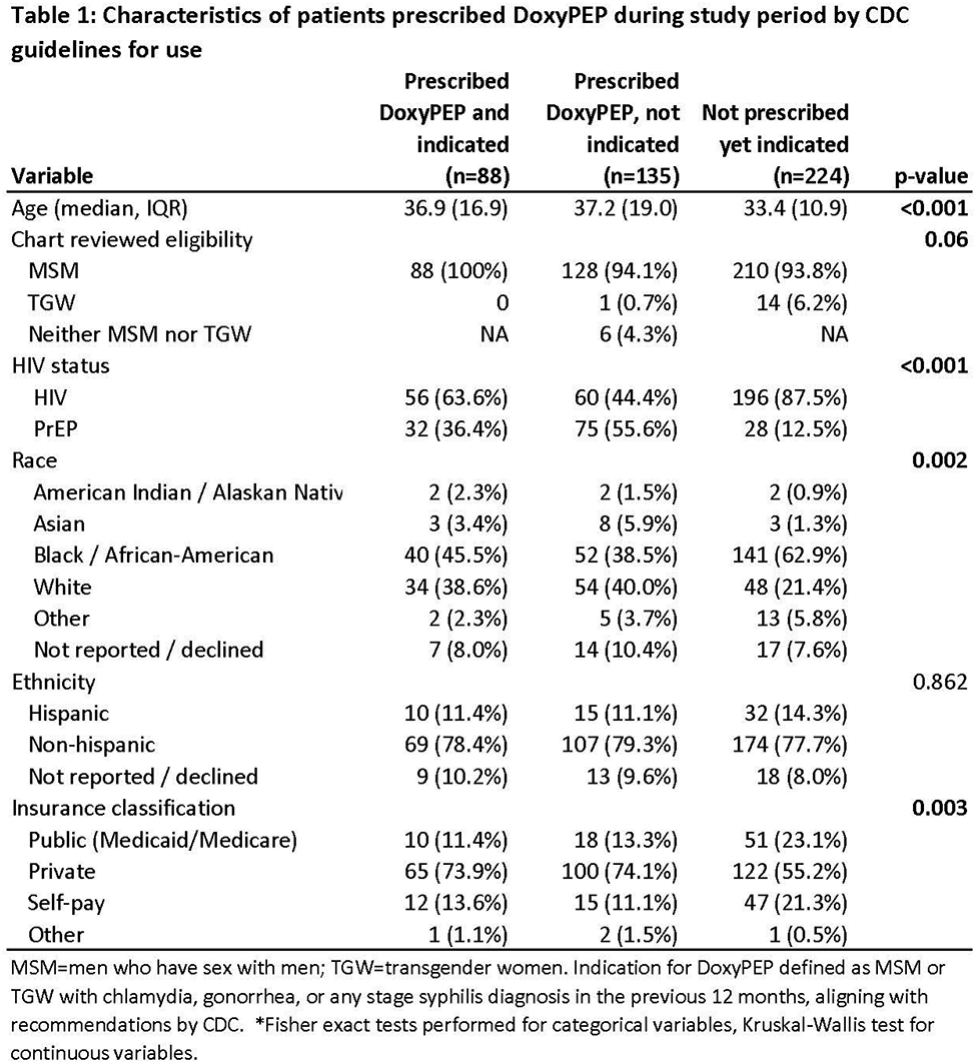
Characteristics of patients prescribed DoxyPEP during study period by CDC guidelines for use

**Methods:**

Patient demographics, STI testing results, encounter information, and DoxyPEP prescription records were abstracted from an online data warehouse for patients seen at an academic HIV/PrEP clinic between 07/01/2023 and 12/31/2024. STI history was assessed to identify patients with an indication for DoxyPEP based on CDC guidelines, with adjudication by clinician chart review. Patient characteristics were compared across three groups: 1) prescribed and indicated, 2) prescribed without indication, 3) indicated yet not prescribed.

**Results:**

There were 223 patients prescribed DoxyPEP during the study period, 88 (39%) of whom met the guidelines for use. Patients on PrEP accounted for 48% of patients prescribed DoxyPEP, and PrEP patients were more likely to receive DoxyPEP without an indication compared to persons with HIV (PWH) (Table 1). The median time from STI diagnosis to DoxyPEP prescription was 89 days (IQR 3-239). Of the 224 MSM or TGW with a STI during the study period who were not prescribed DoxyPEP, a disproportionate share were PWH, Black individuals, and those on public insurance or self-pay coverage. Seventy-five percent of eligible patients had at least one encounter after STI diagnosis in which DoxyPEP was not prescribed.

**Conclusion:**

In an enumeration of early implementation of DoxyPEP into clinical practice, numerous missed opportunities for employing this intervention were identified, particularly among persons with HIV. Continued prospective observation in real-world practice is necessary to better understand emerging trends in DoxyPEP utilization and to ensure prompt identification of inequities as they surface.

**Disclosures:**

All Authors: No reported disclosures

